# Secretory Tissues and Volatile Components of Disc Florets in Several Wild *Helianthus* L. Species

**DOI:** 10.3390/plants13030345

**Published:** 2024-01-24

**Authors:** Jelena Jocković, Nemanja Rajčević, Lana Zorić, Milan Jocković, Aleksandra Radanović, Sandra Cvejić, Siniša Jocić, Ljubodrag Vujisić, Dragana Miladinović, Vladimir Miklič, Jadranka Luković

**Affiliations:** 1 Institute of Field and Vegetable Crops, Maksima Gorkog 30, 21000 Novi Sad, Serbia; aleksandra.radanovic@ifvcns.ns.ac.rs (A.R.); sandra.cvejic@ifvcns.ns.ac.rs (S.C.); sinisa.jocic@ifvcns.ns.ac.rs (S.J.); dragana.miladinovic@ifvcns.ns.ac.rs (D.M.); vladimir.miklic@ifvcns.ns.ac.rs (V.M.); 2 Faculty of Biology, University of Belgrade, Studentski trg 16, 11000 Belgrade, Serbia; nemanja@bio.bg.ac.rs; 3 Faculty of Sciences, Department of Biology and Ecology, University of Novi Sad, Trg Dositeja Obradovića 2, 21000 Novi Sad, Serbia; lana.zoric@dbe.uns.ac.rs (L.Z.); jadranka.lukovic@dbe.uns.ac.rs (J.L.); 4 Faculty of Chemistry, University of Belgrade, Studentski trg 12–16, 11000 Belgrade, Serbia; ljubaw@chem.bg.ac.rs

**Keywords:** disc florets, nectary, plant anatomy, plant micromorphology, VOCs

## Abstract

Although flower pollinator interactions are known to be mediated by floral traits, not enough attention has been paid to the research of secretory tissues and volatile components of sunflower disc florets as potentially important parameters in breeding programs. (1) To our knowledge, this is the first integrated study aimed at better understanding the attractiveness of sunflower capitula to insects. In the study, we have made a very detailed comparative analysis of secretory tissues and the characterization of the volatile components (VOCs) of disc florets in 10 wild perennial *Helianthus* species. (2) For anatomical analyses, cross-sections were obtained from the nectary zone of disc florets using a cryotechnique procedure. Micromorphological observation and morphological and anatomical analysis of disc florets were performed using light and scanning electron microscopy. For VOCs, we applied headspace, GC-FID, and GC/MS analyses. (3) The obtained results indicate that there is a difference between the analyzed traits among studied species. *H. eggertii*, *H. hirsutus*, *H. mollis*, *H. resinosus*, and *H. tuberosus* had high disc diameter values, a high cross-section area and disc floret corolla length, as well as the largest cross-section area and thickness of the disc florets nectary. In the analyzed VOCs, 30 different compounds were detected. The highest yield and quantity of α-Pinene was observed in *H. mollis*. (4) Inflorescence features, such as receptacle diameter, corolla and secretory tissue properties, and floret VOCs production and characterization, provided valuable information that can be used as guidelines in sunflower breeding programs to maximize pollinator attractiveness and increase seed yield.

## 1. Introduction

The genus *Helianthus* consists of various annual (14) and perennial (39) species [1], of which the cultivated sunflower (*Helianthus annuus* L.) is certainly the most widely known. In this regard, unlike many other field crops, cultivated sunflower has at its disposal a large number of wild sunflower species that represent an important gene pool of valuable traits such as tolerance to different abiotic and biotic stresses [2,3], allowing it to progress as an important industrial plant species. Sunflower is an allogamous plant species and one of the most pollination service-dependent for seed production. For the production of hybrid seeds, where pollen has to be transferred from male fertile to male sterile lines, pollinators are essential [4]. Insufficient pollinator attractiveness can result in low seed yields. In food production, pollination has a double role because it affects both the quantity and quality of crops [5]. Honey bees (*Apis mellifera* L.) are the most numerous and most important pollinators in sunflower hybrid seed production and often show distinct preferences for certain sunflowers [6,7,8]. Bees use visual and olfactory information as indicators of floral reward [9]. Pollinator preferences in cultivated sunflowers have been associated with traits such as floret size and color, pollen availability [8], nectar composition or content, and floral odor [10].

There is a negative correlation between the length of the disc florets and the frequency of pollinator visits: longer florets present a physical barrier to a pollinator reaching the nectar reward [8,11]. In a study by Deguardo and colleagues [12], flower size affected pollinator visits, as well as the size of the pollinator, i.e., the species. Waddington and Herbst [13] indicated that the mean functional length of the honey bee proboscis is approximately 7.0 mm. The sunflower florets with larger quantities of nectar sugar and shorter corollas receive greater pollinator services [8]. The “throat diameter” (size of the opening to access nectar) was also positively associated with honey bee visits to highbush blueberry [14]. Sammataro and colleagues [15] and Kamler [16] pointed out that accessibility to nectar, determined by floret length, is a genetically controlled trait that is the most important for sunflower attractiveness to pollinators. Besides genetics, Atlagić and colleagues [17] found that the environment also had a significant influence on the expression of sunflower corolla length.

As a source of food, nectar has an important role in attracting pollinators and improving the percentage of fertilization in sunflowers. Therefore, variation in nectar quality has the potential to influence pollinator preference [18,19]. The nectaries of the Asteraceae family have reached a high level of evolutionary development and, in most cases, are characterized by uniformity in terms of topography and location [20,21]. In a large number of species of the Asteraceae family, nectaries are present only within the disc florets; meanwhile, in lingual florets, they are absent or rarely observed. Therefore, the primary role of disc florets is considered to be attracting pollinators. Sunflower disc florets are epigynous. The corolla is composed of five fused petals. Within the corolla, five anther filaments are attached to the base of the corolla. Nectaries are positioned around the style base of the disc floret on top of the inferior ovary [15]. Sulborska and Weryszko-Chmielevska [22] stated that the qualitative and quantitative characteristics of nectaries can vary both at the interspecies and intraspecies level. Variability can be caused by several factors such as inflorescence size, floret position, microclimatic conditions within the inflorescence, environmental conditions, pollinator activity, soil type, chemical properties, etc. [23,24,25].

Plants produce and release a vast array of volatile organic compounds (VOCs). These compounds play a significant role in plant–environment interactions. Based on the metabolic pathway, plant VOCs can be separated into three groups: terpenoids, phenylpropanoids (benzoids), and various aliphatic hydrocarbons, mostly derived from fatty acids [26,27]. In the pollinator–plant interaction, VOCs provide an olfactory clue that guides insects toward florets in general and nectaries in particular [27,28,29]. Research on honey bees and bumble bees (*Bombus terrestris*) has shown that these insects learn odors faster and can more precisely discriminate between flowers [27]. Bahmani and colleagues [30] found that VOCs are highly diverse among wild *Helianthus*, indicating the value of this genus as a rich genetic resource of traits that could be used for cultivated sunflower improvement. *A. melifera* can detect pollen and nectar in flowers via scent [31,32,33]. While VOCs can be found in different plant tissues, those associated with the flower region are considered to be the most important in plant–pollinator interactions. Most flowers produce VOCs only in corolla; however, VOCs can be found in stamens, pistils, or even pollen grains [34]. Since these compounds can be very toxic to plants, they are often stored in vacuoles, specialized (non-living) cells, or excreted outside the cells [35].

In addition to pollinators, sunflower also attracts plant-feeding insects. The sunflower moth *Homoeosoma electellum* (*Pyralidae*) is, generally, the most widespread and damaging sunflower insect. The larvae of the sunflower moth feed on sunflower pollen, which significantly decreases the success of fertilization. For the management of sunflower moths, there are two well-known plant defenses. The first is secondary plant compounds found in sunflower glandular trichomes, and the second is the presence of phytomelanin in pericarp [36]. Previous in vitro studies have shown that secondary metabolites such as the sesquiterpene lactones of capital glandular trichomes in contact with sunflower moth larvae cause a high death rate [37,38], as well as that flower VOCs in *Helianthus*, together with sesquiterpene lactones, can also have repellent activity on the red sunflower weevil.

In sunflowers, in order to maximize seed yield and production, it is important to understand which traits affect its attractiveness to pollinators and work on their further improvement. Wild relatives have a long history of being used as a source of genes and traits for cultivated sunflower improvement [39]. The progress of the sunflower as an industrial plant species was and still is largely dependent on its wild relatives as a source of desirable variation. However, to our knowledge, detailed data on the comparative micromorphological characteristics of disc flowers and anatomical analyses of the nectar of the wild *Helianthus* species are not available. One of the reasons why studies of this type are very rare is the limited access to collections of wild sunflower species characterized by very small inflorescences with a small number of disc florets, compared to cultivated sunflowers. Most of the phytochemical research on sunflowers was conducted concerning sesquiterpene lactones, a group of non-volatile terpenoids. Taking into account the importance of the genetic variability of wild sunflower species, information on secretory tissues in disc florets can also be useful for breeding cultivated sunflowers to enhance their attractiveness to pollinators [2,3,40]. The same stands for VOCs, for which substantial diversity across the genus was found, indicating the high value of wild *Helianthus* for VOC improvement in cultivated sunflowers [30].

In this paper, we present the first integrated study aimed at obtaining more information about the attractiveness of the capitula to pollinators, specifically, in the genus *Helianthus.* The aim of this study was to define potential useful features for pollinator attractiveness enhancements of the disc florets of ten perennial *Helianthus* species for future breeding programs of cultivated sunflowers by conducting (1) a comparative micromorphological analysis of the basal, apical corolla parts, and anther zone of disc florets; (2) a comparison of the disc florets in the nectary zone; (3) an analysis of the quantity and composition of flower VOCs.

## 2. Results

### 2.1. Morphological Analysis of the Reproductive Region Parts

**Figure 1:**
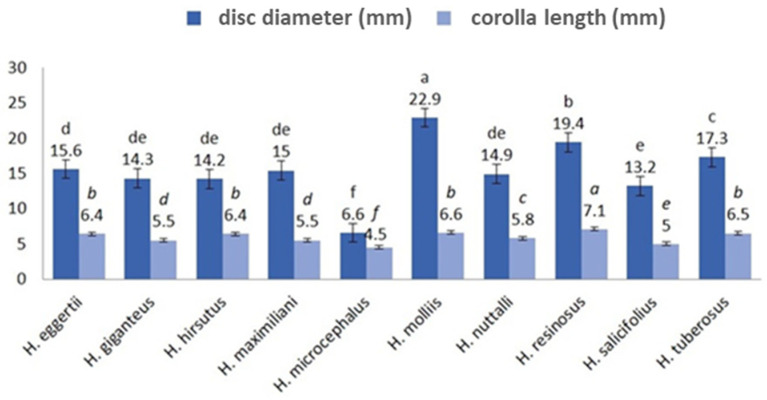
Average values of disk diameter and corolla length in the analyzed *Helianthus* species. Duncan test: values marked with the same letter are not significantly different (the level of significance *p* ≤ 0.05).

In all analyzed species, disc florets were epigenous with corolla composed of five fused petals and free at the top. Significant species heterogeneity was present in the corolla length. *H. resinosus* corolla was significantly longer (7.1 mm) than in other analyzed species. According to the Duncan test, *H. eggertii*, *H. hirsutus*, *H. tuberosus*, and *H. mollis* were grouped together as they were characterized by corolla lengths from 6.4 mm to 6.6 mm (Figure 1). *H. mollis*, *H. resinosus*, and *H. tuberosus* had the widest values in disc diameter (Figure 1). The shortest corolla was observed in *H. microcephalus* (4.5 mm) and *H. salicifolius* (5.0 mm), which also had the lowest values in disc diameter (6.6 mm and 13.2 mm, respectively) (Figure 1).

### 2.2. Micromorphological Characterization of Corolla and Anther Apical Part in Disc Florets

**Figure 2:**
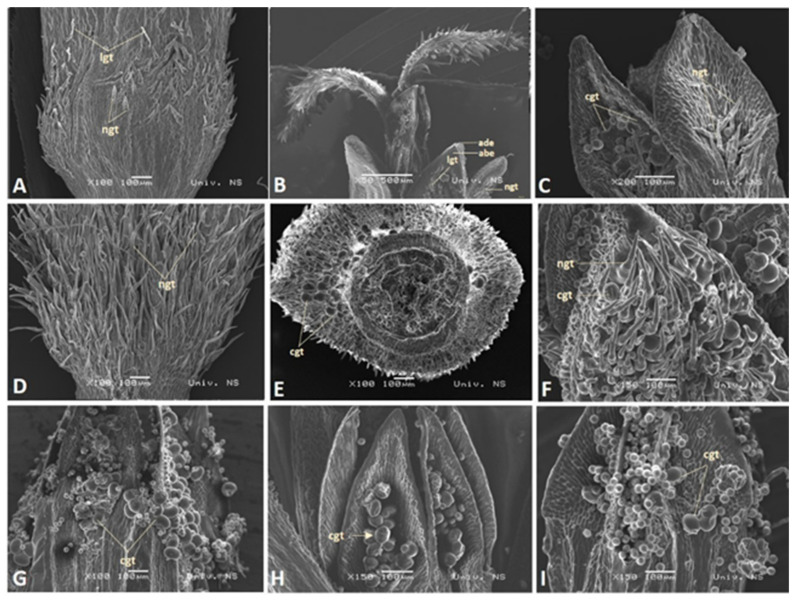
Micrographs of disc florets: Non-glandular trichomes in basal (**A**), apical (**B**), and anther (**C**) zone—*H. microcephalus*; Non-glandular trichomes in the basal zone (**D**)—*H. tuberosus*; Capitate glandular trichomes in basal (**E**) and apical (**F**) zone—*H. mollis*; capitate glandular trichome density in anther top: dense (**G**)—*H. mollis,* medium (**H**)—*H. maximiliani*, and rare (**I**)—*H. tuberosus*; ngt—non-glandular trichomes; ade—adaxial conical epidermal cells; abe—abaxial flat epidermal cells; lgt—linear glandular trichomes; cgt—capitate glandular trichomes.

**Figure 3:**
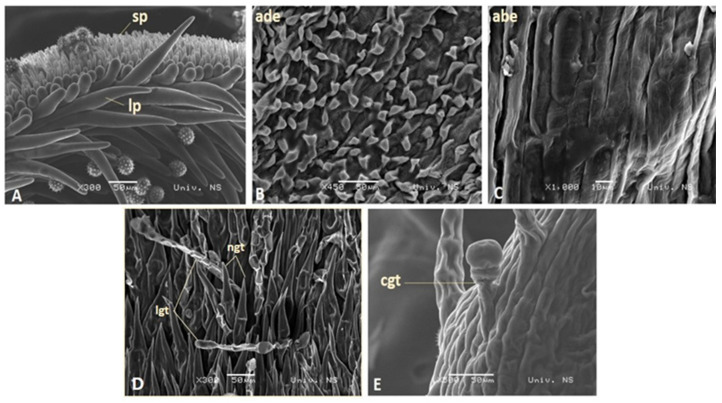
Details of disc florets surface: Stigma lobe (sp—short papile, lp—long papile)—*H. microcephalus* (**A**); Adaxial conical epidermal cells (ade)—*H. microcephalus* (**B**); Abaxial flat epidermal cells (abe)—*H. microcephalus* (**C**); Non-glandular trichomes (ngt) and linear glandular trichomes (lgt)—*H. hirsutus* (**D**); Capitate glandular trichomes (cgt)—*H. mollis* (**E**).

Inside the corolla, five anthers were fused, with free filaments being attached to the corolla tube. The style, with a bi-lobed stigma, was located inside the anther tube (Figure 2B). The abaxial side of the mature stigma was covered with short conical papillae (sp) while the adaxial stigma side was layered with elongated papillae (lp) of various lengths (Figure 3A). In all analyzed species, the abaxial epidermis cells (abe) of the corolla surface were flat, rectangular with smooth ornamentation, and elongated to the longitudinal floret axis (Figure 3C). Unlike the abaxial one, the adaxial epidermis (ade) of the corolla floret contained only conical cells in all analyzed species (Figure 3B).

SEM analysis revealed the presence of multicellular, uniseriate, non-glandular trichomes (ngt), as well as multicellular glandular trichomes (capitate—cgt and linear—lgt) on disc florets. The presence, distribution, and abundance of these trichome types are given in Table 1 for each species (Table 1, Figure 2).

Non-glandular trichomes consisted of basal cells and 1-3 elongated terminal cells (Figure 3D). They were quite varied in length (Figure 2A,D). In all of the examined species except *H. salicifolius*, uniseriate non-glandular trichomes were distributed in all analyzed flower zones (Table 1). They were the densest in the basal zone (Figure 2A,D), then in the apical zone of the corolla (Figure 2B), and sparce at the tips of the fused anther tubes (Figure 2C). Only in *H. salicifolius* were non-glandular trichomes not observed in the anther zone (Table 1). A dense indumentum composed of slightly longer, uniseriate non-glandular trichomes distributed over the basal and apical zone of the corolla was observed only in *H. tuberosus* (Figure 2D).

Linear glandular trichomes are uniseriate structures that are built of several linearly arranged cylindrical cells (Figure 3D). In all species, linear glandular trichomes were present in the basal and apical zones of the corolla (Figure 2A,B). This type of trichomes was not detected in the anther zone (Table 1).

Capitate glandular trichomes were composed of a very short biseriate, multicellular stalk, and round unicellular secretory head (Figure 3E). In contrast to the linear trichomes, the presence of the capitate glandular trichomes in the inner apical part of the anther was observed in all analyzed species (Table 1). Based on the capitate glandular trichome density in the apical part of the anther, the species were classified into three groups: dense, medium, and rare (Figure 2G,H,I). The majority of species had a medium cgt density regarding anthers (Table 1, Figure 2H). Only *H. mollis* possessed densely distributed capitate glandular trichomes over the entire surface of the apical part of the anther (Figure 2G). In *H. mollis*, capitate glandular trichomes were also observed on the outer epidermis of the basal and apical zone of the corolla, where they were very numerous (Figure 2F). The capitate glandular trichomes in the basal zone of the corolla were also present in *H. maximiliani* and *H. resinosus*, although were substantially rarer than in *H. mollis* (Figure 2E; Table 1).

**Figure 4:**
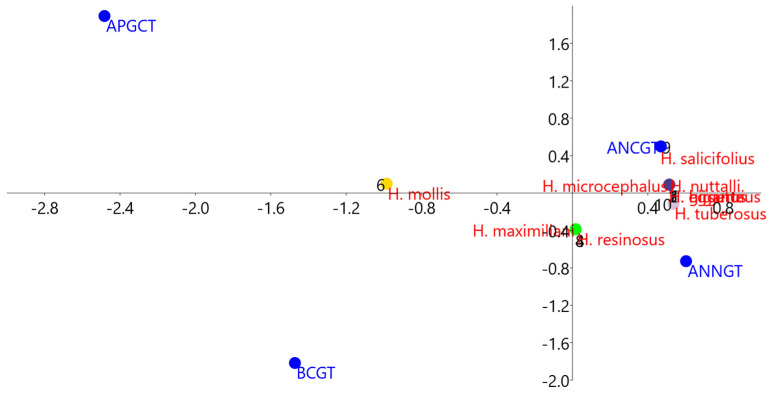
Results of a correspondence analysis (CA), showing the positions of the centroids of the *Helianthus* species in the space of the first two axes.

The results of the correspondence analysis (CA) performed on the micromorphological characters are given in Table 2. A graphical representation of CA showed that *H. mollis* was positioned in the negative part of the first axis as the only species that had capitate glandular trichomes on the outer epidermis of the apical part of the disc florets corolla. *H. mollis*, *H. maximiliani*, and *H. resinosus* were characterized by the presence of capitate glandular trichomes on the basal zone of the corolla due to which the centroids of those species were slightly separated from the homogeneous group comprising the other *Helianthus* species. The parameters of cgt in the anther zone did not contribute significantly to the separation of the species, and neither did the parameters of ngt and lgt, which were the same in all examined species (Figure 4).

### 2.3. Anatomical Analysis of Disc Florets in Nectary Zone

**Figure 5:**
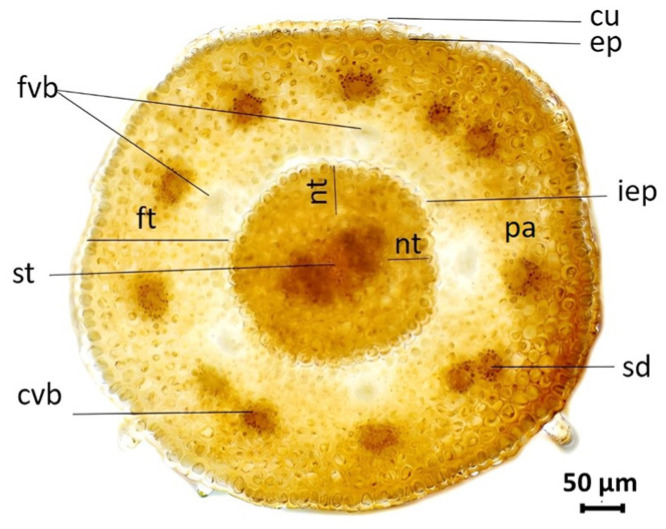
Transverse section through the nectary zone of the disc florets (*H. eggertii*): cu—cuticle, ep—epidermis, pa—parenchyma, iep—inner epidermis, sd—secretory duct, fvb—filaments vascular bundles, cvb—corolla vascular bundles, st—style, nt—nectary thickness, ft—floral tube.

**Figure 6:**
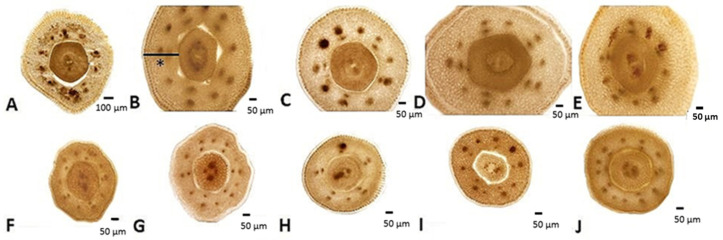
Transverse sections through the nectary zone of the disc florets: (**A**) *H. eggertii*; (**B**) *H. hirsutus*; (**C**) *H. mollis*; (**D**) *H. resinosus*; (**E**) *H. tuberosus*; (**F**) *H. giganteus*; (**G**) *H. nuttalli*; (**H**) *H. maximiliani*; (**I**) *H. microcephalus*; (**J**) *H. salicifolius*. * corolla fused with filaments.

In all ten analyzed species, general disc florets anatomy in the nectary zone was uniform (Figure 5 and Figure 6). The cross-section of the disc florets in the region of the nectaries was round or slightly wavy (in this flower part, the corolla has fused with the filaments). In the transverse section, the epidermis of the corolla was uniseriate, covered by the thick cuticle, and larger than the cells of the inner epidermis. Between the two epidermises, parenchyma tissue was present. Floral tube parenchyma was comprised of thin-walled, densely packed cells arranged in 10–15 layers. In the part closer to the nectary is the presence of usually five collaterally closed vascular bundles, arranged in a circle, belonging to filaments. Above the vascular bundles of filaments, there are vascular bundles that belong to the corolla. Small secretory ducts were present above the phloem tissues of the corolla (Figure 5). Slightly larger secretory ducts were observed in the *H. mollis.*

**Figure 7:**
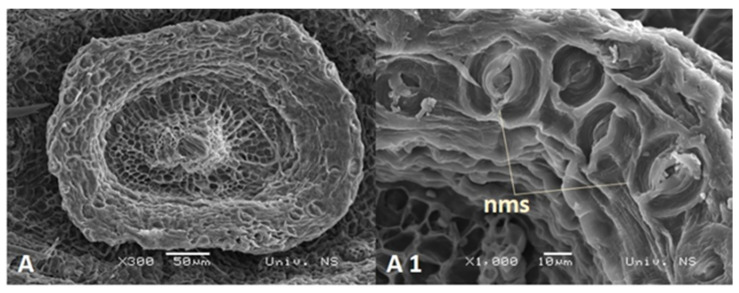
Scanning electron micrographs of nectary surface in *H. maximiliani* (**A**). nms—nectary modified stomata (**A1**).

Nectaries are a tubular structure at the top of the inferior ovary. They were symmetrical, circular, or pentagonal in shape and surrounded the base of the style (Figure 7A). Nectary tissues differentiated into epidermal and parenchymal layers of cells. The epidermis consisted of a single layer of round, densely packed cells covered with cuticles. Nectar is secreted through modified stomata located at the same level as the other epidermal cells (Figure 7A1). The parenchymatic secretory cells of the nectary were localized subepidermally and consisted of 5–8 layers of secretory cells. No vascular bundles were found in the nectar parenchyma. In the center of the flower, a style with two centrally placed vascular bundles was clearly visible (Figure 5).

The mean values of the measured anatomical parameters of the examined florets are summarized in Table 3. Regarding the cross-section area of the corolla (fused corolla with filaments) in the nectary zone, the results showed that *H. eggertii* (1.0 mm^2^), followed by *H. resinosus* (0.9 mm^2^), *H. hirsutus* (0.8 mm^2^), and *H. tuberosus* (0.8 mm^2^), had a higher cross-section area of corolla compared to other examined species. The smallest corolla cross-section areas were recorded in *H. giganteus* and *H. salicifolius* (0.3 mm^2^) (Table 3). There were no statistically significant differences between the species based on the cross-section area of the style. Only *H. eggertii* stands out, with the highest value of this parameter in relation to the other analyzed species. However, the Duncan test revealed statistically significant differences between species in terms of nectar tissue parameters. The largest cross-section area and thickness of nectary tissue were observed in *H. eggertii* and *H. resinosus.* On the other hand, *H. giganteus*, *H. maximiliani*, *H. microcephalus*, *H. nuttallii*, and *H. salicifolius* were grouped together as they were characterized by the smallest nectary area and nectary thickness.

**Figure 8:**
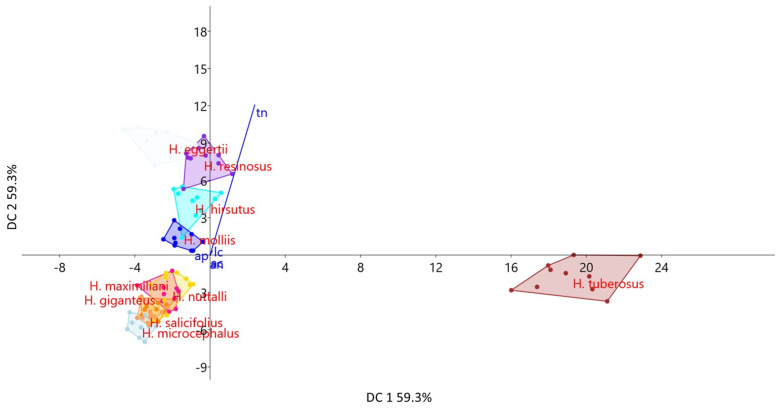
Scatter plot obtained using DCA and the position of centroids in the space of two discriminant axes, based on the disc florets morpho-anatomical characters of the studied *Helianthus* species.

The results of the discriminant component analysis (DCA) performed on the morpho-anatomical characters are given in Table 4. Characters that contributed the most to the discrimination among the species and defined the first two discriminant axes (95.5% of total discrimination) were nectary thickness and corolla length (Table 4, Figure 8). Based on the characters of the first two discriminant axes, species *H. giganteus*, *H. nuttalli*, *H. maximiliani*, *H. microcephallus*, and *H. salicifolius* were grouped together in the negative zone of the graph with significantly lower values of the above-mentioned properties.

### 2.4. Volatile Organic Compounds Analysis

In analyzed flower VOCs, 30 compounds were detected and identified, representing 98.7–100.0% of the total VOC composition (Table 5). In most of the samples, *ɑ*-pinene was the predominant VOC (59.5–95.0%), except for *H. maximiliani* (41.6%), *H. resinosus* (42.2%), and *H. nuttalii* (54.7%). In the *H. maximiliani* and *H. resinosus* species, *ꞵ*-pinene was also present in a high percentage (43.3% and 28.6%, respectively); meanwhile, *ꞵ*-pinene and an unknown hydrocarbon were highly abundant compounds in *H. nuttalii*. In all samples, monoterpenes hydrocarbons were present in a high percentage, and other groups of terpenes presented with less than 20%. Interestingly, *H. mollis* differed strongly from all others based on the strong dominance of *ɑ*-pinene (95.0%), with only a few other compounds being found to have a relative abundance of over 1%.

**Figure 9:**
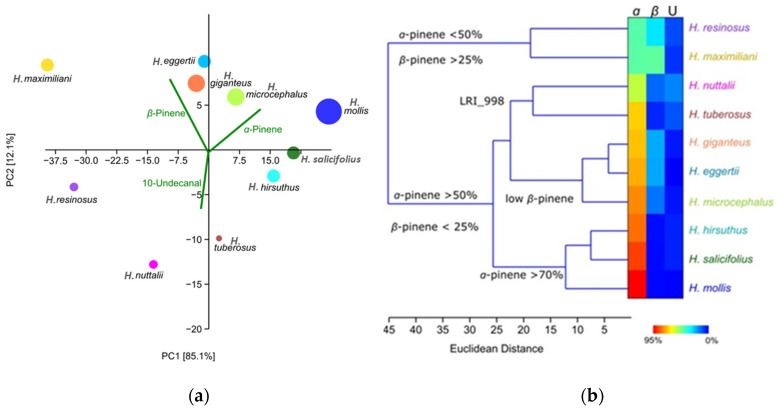
(**a**) PCA scatter plot based on the variability of 30 compounds detected in flower VOCs of *Helianthus* species. The size of spheres reflects relative VOC yields. (**b**) HCA dendrogram based on the relative abundance of 30 compounds detected in flower VOCs of *Helianthus* taxa. The relative abundances of three main compounds, *α*-pinene (α), *β*-pinene (β), and unknown hydrocarbon (U), are presented in the heatmap.

When comparing the relative quantity of VOCs in the samples, once again, *H. mollis* differed from all others by producing 4 to 96 times more volatile compounds in the flower region. Based on the quantity of the VOCs produced, all samples can be separated into four groups: *H. mollis*, with the highest VOCs quantity; *H. microcephalus* and *H. giganteus*, with moderate VOC production; *H. eggertii*, *H. salicifolius*, *H. maximilianii*, and *H. hirsuthus*, with a low production; and *H. resinosus*, *H. tuberosus*, and *H. nuttalii*, with VOCs in traces (Figure 9a). PCA and HCA analyses were performed to assess the differentiation of samples based on VOC variability (Figure 9). When raw values were used, the first two eigenvectors explained 97.2% of the total variability. Three compounds were responsible for separating taxa into four groups, including *ɑ*-pinene, *ꞵ*-pinene, and unknown hydrocarbon. *H. maximiliani* and *H. resinosus* were separated from all others through the low quantity of *ɑ*-pinene and higher abundance of *ꞵ*-pinene. The remaining three groups were formed based on the quantity of *ɑ*-pinene, low quantity of *ꞵ*-pinene, and higher quantity of unknown hydrocarbon (a non-terpenoid compound) (Figure 9b). It is important to underline here that unknown hydrocarbon, along with other aliphatic hydrocarbons, comes from the fatty acid metabolic pathway and not the terpenoid pathway; therefore, a different set of genes is responsible for its production.

**Figure 10:**
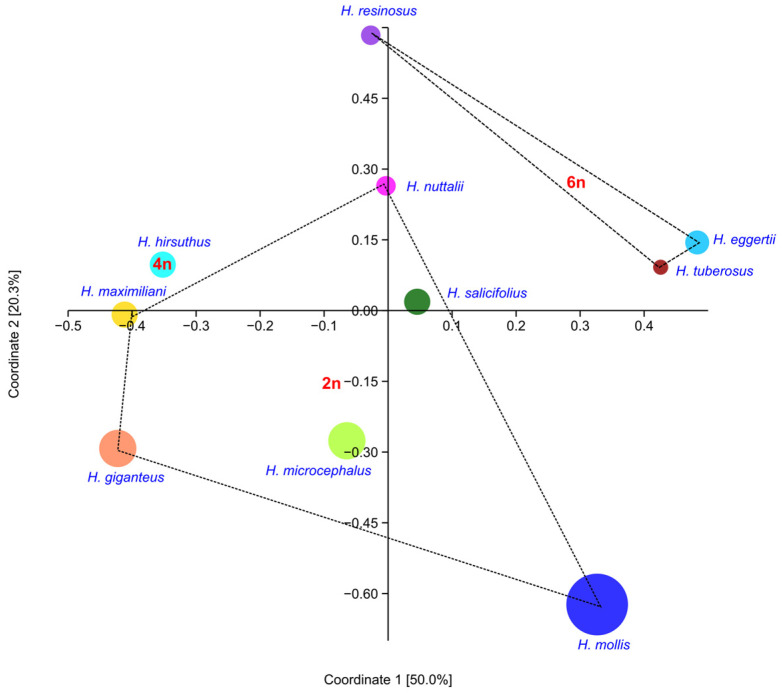
Principle coordinates analysis (Dice distances) based on the presence/absence matrix of 30 flower VOCs from *Helianthus* taxa. The sizes of the spheres represent relative VOC yield.

While the number of VOCs in analyzed samples was significant, environmental factors and storage could affect the quantity of some of the more volatile compounds; therefore, we also looked into the presence/absence matrix for compounds present above 0.1%. Principle coordinate analysis (PCoA) using Dice distances showed different groups compared to the raw data (Figure 10). These groups correlated to the level of ploidy. Based on the presence/absence matrix, tetraploid *H. hirsutus* showed more similarity to some of the diploid taxa than with the hexaploid ones.

**Figure 11:**
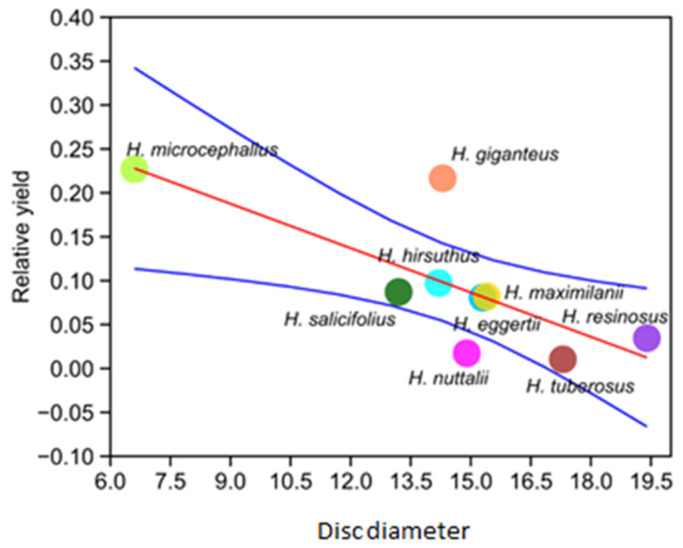
Linear regression analysis of disk diameter and relative yield of VOCs—linear regression curve (red line) with CI95 (blue lines).

Since the size of the nectaries was positively correlated with the size of the flower, we tested if disc diameter is a good predictor of the relative yield of the VOCs. Linear regression did not show a statistically significant relationship when all species were analyzed. However, when *H. mollis* was excluded from the analysis (due to its high production of VOCs and all analyzed morphological characters), a statistically significant negative correlation was found between flower disk diameter and VOC yield (r = −0.7, r^2^ = 0.6, *p* = 0.02). In other words, florets that had bigger nectaries did not produce as many VOCs as the florets with smaller nectaries, suggesting a possible strategy for increasing pollinator attractiveness (Figure 11).

Calculated coefficients of correlation (Pearson) are presented in Table 6. The highest correlation was observed between the thickness of the nectary and the cross-section area of the nectary, as well as between the thickness of the nectary and the cross-section area of the corolla. A high and positive relationship was also observed between disc diameter and corolla length, the cross-section area of the corolla and corolla length, and between the cross-section area of the corolla and the cross-section area of the nectary.

## 3. Discussion

Considering the variety of parameters examined in this research, the obtained information provides a significant advancement in the better understanding of the micromorphological, morphological, and anatomical features of inflorescence, supplemented with VOCs’ characterization of disc florets, in perennial sunflower species. Aside from providing visual cues, inflorescence morphology monitoring can be a useful screening method for the study of plant attractiveness to pollinators. According to Teuber and colleagues [41] in alfalfa and Campbell and colleagues [42] in *Lotus*, the receptacle diameter is significantly and positively correlated with nectar yield. Our results revealed that the diameter of the disc is strongly and positively correlated with the length of the corolla of disc florets. Concerning these traits, the analyzed species are classified into two groups. In the first group are *H. resinosus*, *H. eggertii*, *H. mollis*, and *H. tuberosus* with large disc diameters and large florets. In the second group, there are species with smaller disc diameters and shorter corolla, as well as smaller cross-section areas of corolla (*H. giganteus*, *H. maximiliani*, *H. microcephalus*, *H. nuttallii*, and *H. salicifolius*). The exception was *H. hirsutus,* which had discs that were smaller in diameter but with relatively large florets on them. Sammataro et al. [43] reported that a reduction in floret length by 2 mm could double the number of wild bee visits to florets, thus improving sunflower–pollinator interaction. However, it should be noted that the size of the floret is closely correlated with the seed size [44]. In comparison with the results of an earlier study by Jocković et al. [45] based on the characteristics of the fruits of the same perennial wild *Helianthus* species, there is a certain similarity with our results, i.e., species with larger florets also have larger fruits. The exception is *H. salicifolius*, which was characterized by a short corolla but significantly longer fruits [45]. Considering the strong correlation between these two traits, information on florets morphology can help sunflower breeders simultaneously select for target seed size while minimizing the length of florets to attract pollinators and improve yield [46].

Knowledge of nectary characteristics in disc florets also contributes to a better understanding of plant–pollinator interactions. Previous research on sunflowers indicated that strong correlations between pollinator preference and nectary size (as well as nectary modified stomata number) are present [15]. Phenotypes characterized by larger nectaries with a greater number of stomata are highly attractive to pollinators. According to our results, wild perennial *Helianthus* species have circular or pentagonal nectaries with distinctly modified stomata, which were located mainly in the top part of the nectary tissue, which is typical in Asteraceae [21]. Our results also suggested that, among the analyzed species, there was a noticeable distinction in the development of nectary tissue (cross-section area and thickness), which was closely correlated with flower size. In this regard, *H. eggertii*, *H. resinosus*, *H. hirsutus*, *H. mollis,* and *H. tuberosus* were distinguished with better-developed nectary tissues compared to the other analyzed species. Previous research by Murell and colleagues [47] showed that variations in peduncle vascular system characteristics in *Lotus* (*Lotus corniculatus* L.) could indicate their different conductivity capacities, which is reflected in differences in nectar yield potential. Consequently, in previous research by Jocković and colleagues [48], a comparative analysis of peduncle vascular tissues of 19 wild perennial *Helianthus* species also separated *H. eggertii*, *H. resinosus*, *H. hirsutus*, and *H. mollis* with remarkably higher cross-section areas of vascular bundles as well as more developed xylem and phloem tissues in peduncle. The concordance of these results could indicate that these species may have higher nectar productivity than the other analyzed species.

In addition to the morphological aspects of inflorescence, the micromorphological characteristics of disc florets are also important for sunflower yield. Disc flower micromorphology variation in *Helianthus* species is manifested in the distribution and density of trichomes. Earlier research indicated that sunflower hybrids have 20% less capitate glandular trichomes when compared with wild sunflower species [49]. The present chemical analysis of flower VOCs corresponds with the available literature results, in which monoterpene hydrocarbons dominated the HS-obtained VOCs of *H. tuberosus* and *H. annuus* florets [31,50]. However, in VOCs obtained from *H. annuus* seeds, eugenol acetate dominated [51], suggesting a possibly different composition based on the plant organ. Compounds present in flower VOCs, especially *ɑ*-pinene, *p*-cymene, *ɑ*-terpinene, linalool, and *δ*3-carene, are known olfactory cues for honey bees [32], while *ɑ*-pinene, *ꞵ*-pinene, limonene, camphene, and bornyl acetate repel some predatory insects, like red sunflower weevil [38]. Interestingly, the species with the highest yield of VOCs (*H. mollis*) also had the simplest chemical composition, where most of the VOCs were comprised of a single compound—*ɑ*-pinene. On the other hand, those with the highest number of relatively abundant compounds showed some of the lowest yields of VOCs (e.g., *H. nuttalli* and *H. resinosus*). The presence/absence matrix of compounds present in VOCs confirms a strong genetic determination of VOCs since all of the species were grown under the same environmental conditions; moreover, differences in VOC composition correlated with the ploidy level. VOCs are toxic compounds that need to be produced and stored safely [52]. Only in Asteraceae can they be stored both in secretory ducts and glandular trichomes [53]; however, they do not always contain the same specialized metabolites, nor do they do it synchronously. With that in mind, the relative yield of the VOCs partially corresponds with the size of the secretory ducts in florets. Therefore, the highest VOC yield was in *H. mollis*, corresponding to many large secretory ducts in the corolla. All other species had a variety of secretory ducts, most with a much narrower lumen. 

The corolla and anthers of *H. mollis* were riveted with numerous capitate glandular trichomes (CGTs), unlike other studied taxa, where CGTs were present only in the basal part of the flower and on the anthers. On the opposite end, *H. tuberosus* florets had CGTs only on anthers and produced the lowest quantity of VOCs (96 times less than *H. mollis*). Since capitate glandular trichomes tend to produce non-volatile compounds [54], this could explain why some taxa that had a higher number of glandular trichomes in florets showed lower VOC yields.

Previous cytochemical analyses in *Helianthus* have shown that, even in the same plant, different specialized metabolites are synthesized and stored in different types of glandular trichomes and on different plant organs. For example, the previous investigations on the chemical composition of LGTs and CGTs in *H. annuus* showed a different chemical composition and anatomy of LGTs and CGTs between florets (anthers) and leaves [53]. Nevertheless, the strong presence of sesquiterpene lactones (STLs) was detected in all glandular trichomes. STLs are a group of non-volatile terpenoid compounds that are instrumental in protecting pollen from some insect predators [55,56,57,58], though this was not the case with the sunflower moth [59]. An analysis of the RNAs present in the *Helianthus* glandular trichomes and a histochemical analysis showed the presence of additional compounds, i.e., flavonoids in the lumen [55,58]. Göpfert and colleagues [55] also noticed that STL production stopped with the opening of the flower; however, glandular trichomes continued to enlarge afterward, indicating the synthesis of additional components in these glands, e.g., monoterpenes, phenolic acids, and flavonoids.

In many other species, there is a clear differentiation between VOCs from the florets and other parts of the plant [53,60,61,62,63]. In many angiosperms, and especially in Lamiaceae, flower VOC synthesis is rapid and strongly correlated with pollination [53,64,65].

The correlation between floret size (nectary size) and VOC production is also interesting. Many plants produce rewards for insect pollinators (i.e., nectar or pollen) and advertise it with copious amounts of VOCs. Others, however, mimic the plants that produce large quantities of nectar, though they offer little or no reward. One of the signaling compounds for pollinators, especially honey bees, is *ɑ*-pinene, which was present in abundance in all of the analyzed species.

## 4. Materials and Methods

### 4.1. Plant Material and Environment Data

The collection of wild sunflower species was cultivated at the Institute of Field and Vegetable Crops located at Rimski Šančevi. The location is characterized by chernozem soil and continental semi-arid to semi-humid climate with an average annual precipitation of 617 mm and a mean annual temperature of 11.0 °C (http://www.hidmet.gov.rs, accessed on 22 February 2023). The inflorescences of ten perennial *Helianthus* species were collected in the full flowering phase during the summer of 2020 (June–September) (Table 1). Considering the complexity of the research and to have comparable results, we decided to collect the samples in the same year and from the same stage of maturity, which, to some extent, limited the number of samples due to the nature of the material (small inflorescence diameters with a small number of disc florets). Disc florets for each analysis (morphological, micromorphological, anatomical, and chemical) were taken separately.

### 4.2. Morphological and Micromorphological Analysis

**Figure 12:**
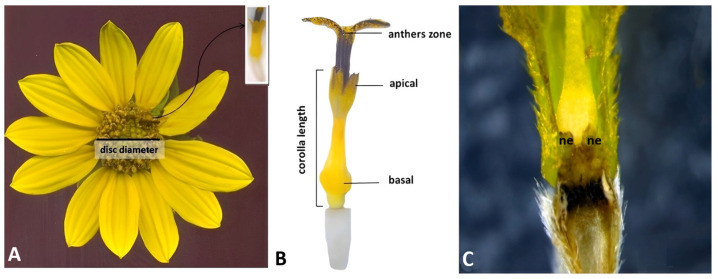
Assessed traits for morphological (**A**,**B**) and micro-morphological analysis (**B**,**C**) of disc florets (*H. eggertii*). ne—nectary.

The disk diameters of five to ten selected central inflorescences of each species were measured with a caliper (Figure 12A). Ten, representative, fresh disc floret samples of each examined species were randomly selected at the same developmental stage (full flowering stage) from the peripheral part of the inflorescence (second open ring). The length of the corolla was measured with a caliper from the base to the apex of the disc flower (Figure 12B). Micro-morphological diversity of disc florets corolla and anther surface was assessed via scanning electron microscopy (SEM). The fresh disc floret of each species was directly observed using a JEOL JSM-6460LV electron microscope (JEOL, Tokio, Japan) at an acceleration voltage of 20 kV”. For additional confirmation on the distribution and type of trichomes, we analyzed the herbarized material wherein florets were directly mounted on a metallic stub using the double adhesive tape and coated with gold for 180 s at 30,144 mA (BAL-TEC SCD005) and then viewed using a JEOL JSM-6460LV electron microscope. Micromorphological characterization of disc florets was performed on basal and apical corolla parts and at the anther zone (Figure 12B). Capitate trichomes density in the anther zone, based on minimum of three disc florets, was determined based on observations made with a scanning electron microscope in the analyzed sample. Based on observation, according to the capitate glandular trichomes density in the apical part of the anther, the species were classified into three groups: dense (densely distributed trichomes in more than 70% of samples), medium (densely distributed trichomes in 50% samples), and rare (rarely distributed trichomes in more than 70% samples).

### 4.3. Anatomical Analysis

For the anatomical analysis of disc florets, ten randomly chosen central inflorescences of each species were selected and fixed in 60% ethyl alcohol. Randomly chosen disc florets were removed from the peripheral part of the inflorescence (second open ring). Light microscopy cross-sections were obtained from the nectar zone of the disc floret (the zone of fusion of the filament and the corolla) using a Leica CM 1850 cryostat at a temperature of −25 °C and at a cutting interval of 30 µm. Semi-permanent slides were mounted in glycerin. Observations and measurements of cross-section areas of corolla (mm^2^), nectary (mm^2^), style (mm^2^), and nectary thickness (µm) were performed using Motic Images Plus 2.0 (Figure 5).

### 4.4. Volatile Organic Compounds Analysis

For volatile organic compounds analysis, only fully opened florets were placed in headspace vials. Plant material was collected from 7 am to 9 am. Several heads (no less than five depending on the size of the inflorescence) were placed in PP zip lock bags and kept in the freezer (−69 °C) until chemical analysis. For static headspace (HS) experiments, ca. 2000 mg of freshly frozen florets were taken from several flower heads from a single individual and placed into 20 mL HS vials. The samples were heated at 90 °C for 15 min under the following procedure: syringe temperature: 100 °C, agitator speed: 500 rpm, fill speed: 100 μL/s, and pullup delay: 1000 ms. Gas chromatography–flame ionization detector (GC-FID) and gas chromatography–mass spectrometry (GC/MS) analyses were carried out with Agilent 7890A apparatus (Agilent, Santa Clara, CA, USA) equipped with a 5975C MSD, FID, and an HP-5MSI fused-silica capillary column 30 m × 0.25 mm × 0.25 μm. For the HS analyses, 2000 μL of generated vapor was extracted from the vial and injected directly into the gas chromatograph using a heated gas-tight syringe. The oven temperature was programmed based on the modified Adams [66] method, rising from 60 °C to 180 °C (3 °C/min), then 180–300 °C (40 °C/min), with a final hold of 5 min; injector: 220 °C; FID detector: 300 °C; carrier gas: He at constant pressure (1.0 mL/min at 210 °C), split ratio, 5:1; EI-MS (70 eV), *m*/*z* range 40 to 550. Identification of all the compounds in the analyses was matched through a comparison of their linear retention indices (relative to C8–C36 *n*-alkanes on the HP-5MSI column) and MS spectra with those of standards from NIST11, Adams4, and homemade MS library databases. The relative contents of the 49 identified compounds were calculated from the FID peak areas. The relative yield was calculated as a percentage of 50 of the total area in relation to the maximal area value obtained in all samples. Retention indices were calculated for all compounds using the following formula: LRI = 100 × (trs − 2 trn)/(trn + 1 − trn) + 100 × n.

### 4.5. Statistical Analysis

To evaluate disc floret characters, multivariate analyses were performed using PAST 4.11 [67]. Means, standard errors, correlations, and coefficients of variation were calculated. The significance of differences in the measured anatomical parameters between the analyzed species was determined using Duncan’s test (*p* ≤ 0.05). DCA—discriminant component analysis was performed to test the hypothesis that the analyzed sample comprises distinct groups. Principal component and linear regression analyses were performed to assess the variability and relationship between data, respectively. Multivariate analyses DCA and PCA are presented in tables and graphically. Qualitative characters were subjected to correspondence analysis (CA) to check the grouping tendency of the examined species. For VOCs, univariate and multivariate analyses were performed. PCA, principle coordinate analysis (PCoA), and hierarchical cluster analysis (HCA) were used to study the relationship between samples, while linear regression analysis and simple correlation (Pearson’s correlation) were used to study correlations within VOCs and between VOCs and morphological data.

## 5. Conclusions

The results obtained in this research provide significant information necessary for a more complete understanding of the micromorphological, morphological, and anatomical features of inflorescences, supplemented with a VOC characterization of disc florets, in perennial sunflower species. To increase pollinator attractiveness through breeding programs, floret traits should be informative and actionable. In our study, some of the inflorescence features have been demonstrated to be especially useful in distinguishing analyzed *Helianthus* species and, therefore, could be of interest for breeding. *H. eggertii*, *H. hirsutus*, *H. mollis*, *H. resinosus*, and *H. tuberosus* differed from other analyzed species as they had high values regarding the disc diameter, cross-section area, and length of the disc florets. Others, however, mimic the plants that produce large quantities of nectar, though they offer little or no reward. One of the signaling compounds for pollinators, especially honey bees, is *α*-pinene, which was present in abundance in all of the analyzed species.

Regarding the flowers corolla cross-section area and the thickness of the disc florets nectary, the highest VOC yield was recorded for *H. mollis.* Bearing in mind that honey bees are the most important sunflower pollinators, the corolla length of *H. eggertii*, *H. hirsutus*, *H. mollis*, *H. resinosus*, and *H. tuberosus* would not represent a physical barrier to pollinator-foraging activities. Thus, the abovementioned species are potentially useful genetic resources for improving sunflower attractiveness to pollinators, as well as seed yield, taking into account the close relationship between the length of the corolla and the length of the fruit (achene).

In sunflower breeding, selected genotypes should, in addition to high attractiveness, possess a certain level of resistance to insects and pests. The presence of trichomes in the zone of disc florets can have an important role in the defense against pests. Thus, an increase in their density, especially of capitate glandular trichomes in the anther zone, can lead to a decrease in sunflower yield loss caused by insects, which should be investigated in future studies. Regarding the development of capitate glandular trichomes in the region of the floret, the *H. mollis* stands out, as its capitate glands are significantly more developed compared to all other analyzed species.

The attractiveness factors examined in this research, such as receptacle diameter, corolla and secretory tissue properties, flower VOC production, and characterization, can be used as indicators of species attractiveness to pollinators in sunflower breeding programs.

## Figures and Tables

**Table 1:** Presence, distribution, and abundance of trichomes on the abaxial epidermis of disc florets corolla and in the inner apical part of the anther in analyzed *Helianthus* species.

Species	Abaxial Corolla Epidermis						Inner Anthers Apical Parts		
	Basal			Apical					
	ngt	lgt	cgt	ngt	lgt	cgt	ngt	lgt	cgt
*H. eggertii* Small	+	+	0	+	+	0	+	−	2
*H. giganteus* L.	+	+	0	+	+	0	+	−	2
*H. hirsutus* Raf.	+	+	0	+	+	0	+	−	2
*H. maximiliani* Schrader	+	+	1	+	+	0	+	−	2
*H. microcephalus* T.&G.	+	+	0	+	+	0	+	−	2
*H. mollis* Lam.	+	+	3	+	+	3	+	−	3
*H. nuttalli* T.&G.	+	+	0	+	+	0	+	−	2
*H. resinosus* Small	+	+	1	+	+	0	+	−	2
*H. salicifolius* Dietr.	+	+	0	+	+	0	−	−	2
*H. tuberosus* L.	+	+	0	+	+	0	+	−	1

Note: ngt—non-glandular trichomes; lgt—linear glandular trichomes (+: detected, −: not detected); cgt—capitate glandular trichomes (0: not detected, 1: rare, 2: medium, 3: dense).

**Table 2:** Correspondence analysis (CA) based on the presence and distribution of non-glandular and glandular trichomes on the abaxial epidermis of corolla and the inner apical part of the anther in the disc florets of studied *Helianthus* species.

Characters	CA 1	CA 2	CA 3
BCGT (Capitate glandular trichomes in basal zone)	−1.472	−1.817	−0.964
APCGT (Capitate glandular trichomes in apical zone)	−2.482	1.892	1.263
ANNGT (Short non-glandular trichomes in anther zone)	0.602	−0.728	1.488
ANCGT (Capitate glandular trichomes in anther zone)	0.4691	0.498	−0.617

Red values represent characters that contributed the most significantly to discrimination among the species.

**Table 3:** Anatomical characteristics of the disc florets of the *Helianthus* species (mean value ± standard error, coefficient of variation CV (%).

Species	Cross-Section Area			Nectary Thickness (µm)
	Corolla (mm^2^)	Nectary (mm^2^)	Style (mm^2^)	
*H. eggertii*	1.0 ± 0.02 ^a^ (7.4)	0.2 ± 0.006 ^a^ (9.7)	0.04 ± 0.003 ^a^ (26.2)	150.9 ± 4.4 ^a^ (9.3)
*H. giganteus*	0.3 ± 0.005 ^g^ (5.4)	0.07 ± 0.001 ^e^ (7.4)	0.01 ± 0.0007 ^d^ (15.8)	75.3 ± 1.9 ^ef^ (8.4)
*H. hirsutus*	0.8 ± 0.03 ^c^ (11.0)	0.1 ± 0.004 ^c^ (10.9)	0.03 ± 0.001 ^c^ (13.8)	111.2 ± 4.2 ^c^ (11.9)
*H. maximiliani*	0.4 ± 0.01 ^e^ (10.1)	0.07 ± 0.005 ^e^ (20.8)	0.02 ± 0.001 ^d^ (25.1)	77.2 ± 2.0 ^ef^ (8.3)
*H. microcephalus*	0.4 ± 0.009 ^fg^ (8.0)	0.06 ± 0.003 ^e^ (19.3)	0.01 ± 0.001 ^d^ (25.9)	74.3 ± 2.6 ^ef^ (11.1)
*H. mollis*	0.6 ± 0.008 ^d^ (4.4)	0.09 ± 0.005 ^d^ (17.5)	0.02 ± 0.001 ^d^ (25.2)	97.3 ± 3.4 ^d^ (11.1)
*H. nuttallii*	0.4 ± 0.02 ^ef^ (16.5)	0.07 ± 0.004 ^e^ (19.7)	0.02 ± 0.001 ^d^ (27.0)	72.9 ± 3.5 ^f^ (15.2)
*H. resinosus*	0.9 ± 0.04 ^b^ (12.5)	0.15 ± 0.002 ^b^ (4.7)	0.03 ± 0.001 b^c^ (10.8)	124.6 ± 3.8 ^b^ (9.6)
*H. salicifolius*	0.3 ± 0.009 ^g^ (8.5)	0.06 ± 0.002 ^e^ (8.7)	0.01 ± 0.0003 ^d^ (6.6)	82.9 ± 2.3 ^e^ (8.8)
*H. tuberosus*	0.8 ± 0.3 ^c^ (12.1)	0.1 ± 0.002 ^c^ (10.7)	0.03 ± 0.001 ^b^ (20.7)	109.1 ± 2.3 ^c^ (6.7)

Duncan test values marked with the same letter are not significantly different (the level of significance *p* ≤ 0.05).

**Table 4:** DCA of quantitative morpho-anatomical characters of disc florets in the studied *Helianthus* species.

Characters	DC 1	DC 2	DC 3
Corolla length (lc)	−0.046	**0.123**	**−0.203**
Cross-section area			
corolla (ac)	−0.016	0.047	0.021
nectary (an)	0.002	0.008	0.006
style (ap)	−0.005	0.003	0.002
Nectary thickness (tn)	**0.893**	**4.537**	**3.909**

Bolded numbers represent characters that contributed the most to the discrimination of species.

**Table 5:** Chemical composition of VOCs obtained from disc florets of *Helianthus* species.

LRI	Compound/Species	E	G	H	Ma	Mi	Mo	N	R	S	T
** Ploidy level		6n	2n	4n	2n	2n	2n	2n	6n	2n	6n
801	3-Methylbutyraldehyde	0.7	2.8	4.7	6.0	2.2	tr	5.5	2.0	6.7	11.4
841	Hexanal	tr	0.8	0.9	1.7	1.2	tr	5.8	1.4	1.2	4.4
901	*cis*-4-Heptenal	-	0.3	0.3	0.7	0.3	-	1.9	0.7	0.5	1.0
922	Tricyclene	tr	tr	tr	tr	tr	tr	-	-	0.1	-
926	*α*-Thujene	tr	0.2	-	0.2	0.1	0.1	-	-	0.2	-
934	*α*-Pinene	72.1	70.6	79.1	41.6	76.1	95.0	54.7	42.2	81.6	59.5
948	Camphene	0.3	0.8	1.4	0.8	1.1	0.8	0.4	0.5	2.3	1.7
953	Thuja-2,4(10)-diene	0.3	-	0.5	-	0.2	0.4	0.3	-	0.3	0.7
973	Sabinene	0.3	0.9	-	1.1	-	0.6	1.0	0.8	-	-
977	*β*-Pinene	21.0	19.8	1.6	40.5	14.4	0.6	12.5	28.8	1.2	4.6
992	*β*-Myrcene	0.8	0.3	4.6	0.4	0.7	0.3	-	2.5	-	1.7
997	2-Pentyl furan	-	0.5	-	0.9	0.4	-	0.9	1.7	0.5	3.7
998	Unknown ***	-	1.5	3.6	3.5	1.7	-	14.1	5.9	3.7	5.0
1008	*δ*-3-Carene	tr	-	-	-	-	tr	-	-	-	-
1020	*α*-Terpinene	-	tr	tr	0.1	tr	tr	-	-	-	-
1028	*p*-Cymene	-	0.1	tr	0.3	tr	tr	0.4	-	tr	-
1030	Limonene	2.7	1.0	2.3	1.6	0.7	1.5	1.4	12.1	1.3	4.9
1064	*γ*-Terpinene	tr	0.1	0.1	0.2	tr	tr	0.2	0.1	-	-
1099	Terpinolene	tr	tr	tr	tr	tr	-	-	0.1	-	-
1123	2,7-dimethyl-2,6-Octadien-1-ol	tr	tr	tr	-	tr	tr	-	-	-	-
1130	*α*-Campholenal	0.4	tr	0.3	tr	0.3	tr	0.4	0.2	0.1	-
1142	*trans*-Pinocarevol	0.1	tr	-	tr	tr	-	-	-	-	-
1148	*cis*-Pinen-3-ol	tr	tr	tr	-	tr	tr	-	-	-	-
1165	Pinocarvone	0.2	tr	tr	tr	tr	tr	-	-	-	-
1199	Myrtenal	0.1	tr	-	tr	tr	tr	-	-	-	-
1288	Bornyl acetate	0.2	tr	-	tr	tr	tr	-	-	0.1	-
1422	(*Z,Z*)-*α*-Farnesene	tr	tr	-	tr	-	tr	-	0.1	-	-
1440	*α-trans*-Bergamotene	tr	tr	tr	-	tr	tr	-	0.3	-	-
1462	Amorpha-4,11-diene	-	-	-	-	0.2	-	-	-	-	-
1483	Germacrene D	0.1	tr	tr	tr	tr	tr	-	0.4	tr	0.1
Total monoterpens		99.0	95.0	91.3	88.8	95.1	99.7	77.1	88.6	88.6	77.5
Monoterpene hydrocarbons		97.9	94.7	90.7	88.6	94.7	99.4	76.7	88.4	88.3	77.5
Oxygenated monoterepenes		1.1	0.3	0.5	0.2	0.4	0.3	0.4	0.2	0.3	0.0
Sesquiterpene hydrocarbons		0.2	0.0	0.1	0.0	0.2	0.1	0.0	0.8	0.0	0.1
Others		0.7	5.0	8.5	11.1	4.6	0.1	22.5	10.3	11.4	21.1
No. of compounds		24.0	26.0	21.0	23.0	27.0	23.0	14.0	17.0	16.0	12.0
TOTAL		99.9	100.0	99.8	100.0	100.0	99.8	99.6	99.7	100.0	98.7
	Relative yield *	8%	22%	10%	8%	23%	100%	2%	3%	9%	1%

* Relative yield calculated as % of the total area on FID in comparison to the highest FID area; tr—<0.1%; - not detected E—*H. eggertii,* G—*H. giganteus*, H—*H. hirsutus*, Ma—*H. maximiliani*, Mi—*H. microcephalus*, Mo—*H. mollis*, N—*H. nuttallii*, R—*H. resinosus* S—*H. salicifolius*, T—*H. tuberosus*. ** taken from Schilling and Heiser (1981). *** unknown compound, *m*/*z* (intensity): 41 (100), 67 (81), 55 (80), 54 (46), 79 (35), 93 (32), 82 (31), 57 (31), 83 (31), 98 (29).

**Table 6:** Pearson’s coefficients of correlation.

	Disc Diameter (mm)	Corolla Length (mm)	C-s Area of Corolla (mm^2^)	C-s Area of Nectary (mm^2^)	Nectary Thickness (µm)	VOCs Yield
Disc diameter (mm)	1					
Corolla length (mm)	0.83	1				
C-s area of corolla (mm^2^)	0.42	0.81	1			
C-s area of nectary (mm^2^)	0.37	0.68	0.89	1		
Nectary thickness (µm)	0.40	0.72	0.95	0.96	1	
VOCs yield	−0.75	−0.66	−0.41	−0.34	−0.39	1

C-s: cross-section.

## Data Availability

Data are available upon request due to restrictions, e.g., privacy or ethics. The data presented in this study are available upon request from the corresponding author. The data are not publicly available due to the significance of the results.
